# Design of a Micro-Electro Mechanical System Quad Mass Gyroscope with Compliant Mechanical Amplification

**DOI:** 10.3390/mi15010124

**Published:** 2024-01-11

**Authors:** Jingchuan Zhou, Dacheng Xu, Xinxin Li, Fang Chen

**Affiliations:** 1 State Key Laboratory of Transducer Technology, Shanghai Institute of Microsystem and Information Technology, Chinese Academy of Sciences, Shanghai 200050, China; zhoujc@mail.sim.ac.cn (J.Z.); xxli@mail.sim.ac.cn (X.L.); 2 University of Chinese Academy of Sciences, Beijing 100049, China; 3 School of Electronic and Information Engineering, Soochow University, Suzhou 215006, China; xudacheng@suda.edu.cn

**Keywords:** MEMS gyroscope, quadruple mass gyroscope, mechanical amplification, large mechanical sensitivity, finite element analysis

## Abstract

In this work, a novel mechanical amplification structure for a MEMS vibratory gyroscope is proposed with the aim of improving their sensitivity. The scheme is implemented using a system of micromachined V-shaped springs as a deflection amplifying mechanism. The effectiveness of the mechanism is first demonstrated for a capacitive fully decoupled quad mass gyroscope. A proof of concept vertical-axis mechanically amplified gyroscope with an amplification factor of 365% has been designed, simulated and fabricated, and results from its evaluation are presented in this paper. Experimental results show that the natural frequency of the gyroscope is 11.67 KHz, and the full scale measurement range is up to ±400°/s with a maximum nonlinearity of 54.69 ppm. The bias stability is 44.53°/h. The experiment results show that this quad mass gyroscope’s performance is a very potential new way of reaching the navigation grade in the future.

## 1. Introduction

Micro-Electro Mechanical Systems (MEMS) and especially gyroscope sensors are part of a flourishing market ranging from consumer electronics to space applications. Although MEMS gyroscopes have been evolving for more than 25 years, there is still considerable research effort for further improving their performance [1,2]. The performance of MEMS gyroscopes is often limited to transducing the initial physical signal into a mechanical displacement or deformation, which in turn causes a change in capacitance or piezoresistive. Typically, the resulting electrical changes are quite small and need to be amplified, which is usually carried out using charge amplifiers or transimpedance amplifiers. However, noise and other disturbance sources at the front-end of the electronic amplifier such as parasitic capacitances as well as intrinsic noise sources of the amplifier itself are enhanced, which restricts the sensitivity and the resolution of gyroscope systems [3,4].

The differential structure designs with two out-of-phase vibrating proof masses to reduce the vibration and acceleration induced errors are reported [5,6,7,8]. A further increase in the rejection of external stimuli has been proposed by University of California (Irvine) with an 8 × 8 mm^2^ quadruple mass gyroscope (QMG), which can minimize frequency and damping mismatches and maximize the Q-factor [9,10,11]. QMG is fully dynamically balanced in forces and momentum and provides mechanical rejection of external vibrations and mechanical shocks along both the drive and sense axis. Moreover, Singapore Institute of Microelectronics presents a QMG with new working modals, which is similar to the wine-glass vibrating gyroscope [12]. The size of this QMG was 5.8 × 5.8 mm^2^. Southeast University proposed a symmetrical quadruple mass gyroscope with four hair masses at 8 × 8 mm^2^. It realized an improvement in sensitivity and a differential detection of the Coriolis force [13]. Tsinghua University designed a QMG in 7.7 × 7.7 mm^2^ with a center support frame which had a similar working principle with ring-like gyroscopes [14]. The QMGs demonstrated above were operated under wine-glass working modes. However, compared to the working modes used in University of California (Irvine), the wine-glass type did not provide a symmetric working mode which should be an advantage of QMGs. It should be noticed that all kinds of QMGs result in a high rejection to external vibrations and shocks [15,16].

Instead of amplifying the pick-off signal in the electrical domain for the first time, we show a primary amplification in the mechanical domain using a new compliant mechanical amplifier. The Coriolis force signal is therefore amplified before electric noise disturbances can affect the analog signal [17]. Compliant mechanical amplifiers are well known for piezoelectric actuator applications. Generally, these amplifiers can be divided into flexure-based and lever-based mechanisms [18,19,20]. In recent studies, lever-type compliant amplifiers had a great combination with an accelerometer. The University of Southampton have proposed a capacitive accelerometer with a lever-type mechanical motion amplifier [21]. By observed the contrast between the change of capacitance from the proof mass and end of the lever, it showed a higher sensitivity without sacrificing bandwidth. The University of California, Berkeley, proposed a resonant accelerometer with a two-stage lever as a force amplifier [22]. All of the mechanisms mentioned above realized a great amplification effect. We innovatively combined the compliant mechanical amplifier with a quadruple mass gyroscope.

The system proposed in this work is a novel rhombus-like compliant mechanical amplification concept for a silicon micro-machined resonant quadruple mass gyroscope (QMG) to improve its performance with a smaller active area of 4 × 5 mm^2^. The scheme is implemented using a system of rhombus-type compliant amplifiers as a deflection amplifying mechanism. This results in a larger Coriolis deflection and a higher signal-to-noise ratio compared to conventional QMGs. It also has the supplementary benefit of reducing the energy leakage from drive mode to sense mode.

The paper is organized as follows: Section 2 gives the demonstration about the structural design of quad mass gyroscopes, which includes the basic structure and the rhombus-like compliant mechanical amplification structure design. The analysis of the amplification structure is briefly introduced. Section 3 confirms the validity of the structural design with Finite-Element Analysis (FEA). Section 4 describes the fabrication process of the quad mass gyroscope, which is based on cavity SOI and gold–silicon eutectic bonding. The experimental methods and results are presented in Section 5. Finally, Section 6 concludes the whole paper with a summary.

## 2. Design and Analysis

### 2.1. Structure and Working Principle

**Figure 1:**
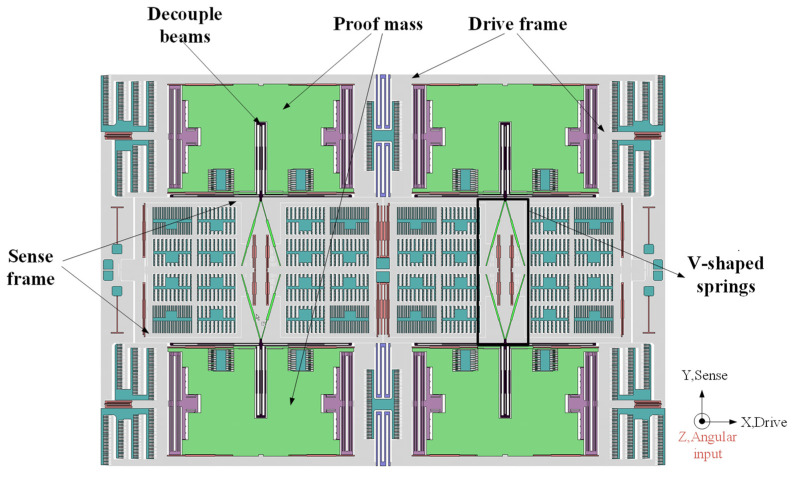
Structure of quad mass gyroscope.

Figure 1 shows the schematic diagram of the mechanical design of our gyroscope. It is a symmetric structure with quad proof masses. Each proof mass is connected to the drive frame by four straight beams. Two masses on both sides are linked together with a U-shaped beam, which can provide a large range of displacement that is linear to the stress. Masses on different sides are connected with a balance leverage which can make sure masses move in opposite direction but at the same amplitude. The upper and lower masses are connected through V-shaped springs, and the other end of V-shaped springs are connected to the sense frame which is composed of the movable parts of comb capacitors. There are four anchors in left and right balance beams, respectively, and two anchors in the middle of this structure.

**Figure 2:**
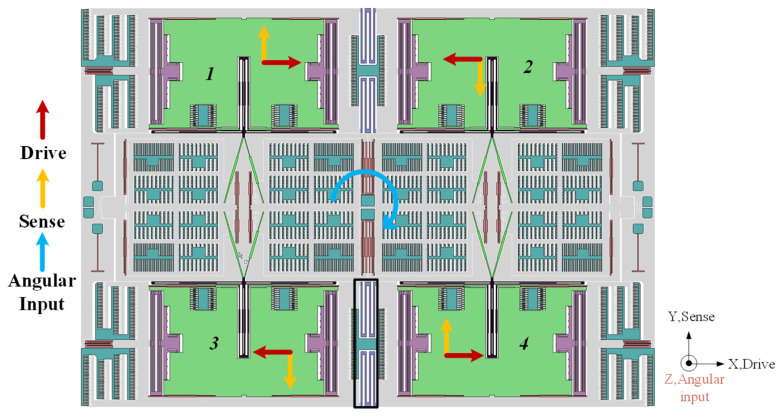
The basic working principle of quad mass gyroscope.

The working principle of the gyroscope is explained as follows. The proof masses are electrostatically actuated to vibrate in the X direction as shown in Figure 2. Four masses were named as 1 to 4. For example, when the gyroscope worked, mass 1 would vibrate in a different direction to mass 2 and mass 3. The movement of each proof mass in the QMG is depicted in red arrows in Figure 2, and that was the basic drive mode of this QMG. When there is a rotation around the *Z*-axis, a Coriolis force would be induced. Then, it would apply on the proof mass to make it move in the Y direction, as the yellow arrows show in Figure 2.

The displacement caused by the Coriolis force is then amplified by V-shaped springs (compliant mechanical amplification structure) which is used as a mechanical motion amplifier. Then, it changed the movement of the sense frame from the Y direction to X direction. Sensing capacitors connected to the sense frame can detect the movement. Because of the mechanical motion amplifier, the displacement is greatly increased. So, the mechanical sensitivity was literally improved. Moreover, the motion coupling of the sense and drive frame is only through the proof mass, the drive and sense frame movement can be perfectly decoupled due to a decouple spring design.

### 2.2. Design Strategy

The Coriolis effect is not obvious in the microscale resonator, and its signal is very weak. Therefore, it is quite necessary to design a gyroscope with high mechanical sensitivity. Mechanical sensitivity is defined as the ratio of the displacement of the sense mode and the input angular velocity. The mechanical sensitivity of QMG can be expressed by (1).

(1) $$y\left(t\right)=\frac{2Q_{x}\omega_{x}A_{drive}\Omega_{z}}{K_{x}\omega_{y}^{2}}\frac{1}{\sqrt{{\left(1-\frac{{\omega_{drive}}^{2}}{\omega_{y}^{2}}\right)}^{2}+{\left(\frac{\omega_{drive}}{Q_{y}\omega_{y}}\right)}^{2}}}\cos\left(\;\omega_{drive}t+\phi_{y}-\phi_{x}\right)$$

In Equation (1), *Q_x_* and *Q_y_* are quality factors of the drive and sense mode. *ω_drive_* is the frequency of the drive force. *ω_x_* and *ω_y_* are the natural frequency of the drive and sense mode. *A_drive_* is the amplitude of the drive mode. *Ω_z_* is the input angular rate. *K_x_* is the stiffness coefficient of the drive mode. When the sense mode is equal to the drive mode frequency, the mechanical sensitivity changes to (2).

(2) $$y(t)=\frac{2Q_{x}Q_{y}A_{drive}\Omega_{z}}{K_{x}\omega_{x}}\sin(\omega_{x}t-\phi_{y})=x_{drive}\frac{2Q_{y}\Omega_{z}}{\omega_{x}}\sin(\omega_{x}t-\phi_{y})$$

It was shown clearly from Equations (1) and (2) that there is a large mechanical sensitivity need mode match, large *Q* value and large drive amplitude [23]. During the design state, the frequency tunning electrodes were reserved to the realized mode match. To obtain a large *Q* value, the fabrication process including eutectic bonding and integrated getter in the cavity of the CAP wafer to ensure a high vacuum level. Moreover, QMG’s drive springs were folded into double U-shape springs which can provide a big movement of up to 20 μm. Despite the fact that the QMG has all of the factors considered above, in this work, we present a mechanical motion amplifier to increase the sensitivity of the Coriolis detection. The sensitivity amplification part was implemented in the front of electronic part, then the desired amplification will be achieved without the generation of additional noise.

### 2.3. Mechanical Motion Amplifier

**Figure 3:**
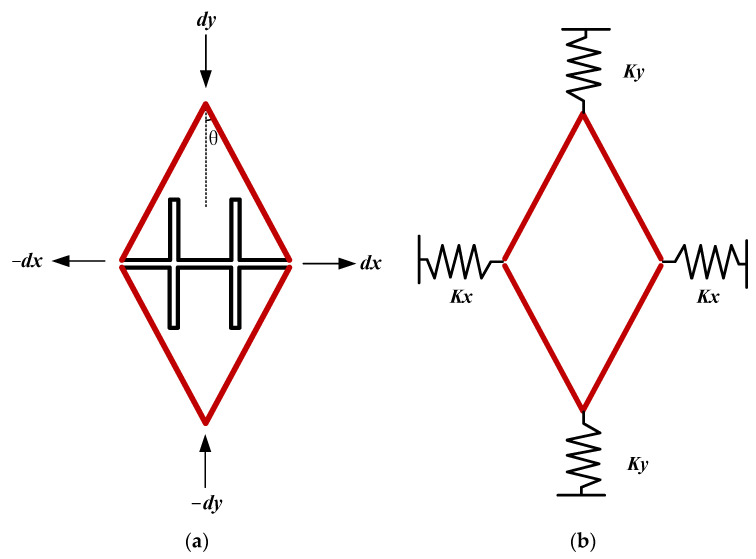
Simplified model of mechanical motion amplifier; (**a**) scheme and working principle of the V-shaped springs; (**b**) the simplified model for stiffness transformation analysis.

Figure 3 shows the proposed design of the Mechanical motion amplifier which is basically simplified to V-shaped springs. As Figure 3 shows, *d_y_* is the proof mass displacement caused by Coriolis force, and $d_{x}$ is the displacement of sense frame, which is also the movement of the sensing capacitors’ movable parts.

The angle *θ* is a parameter, which would decide the amplification factor (from *d_y_* to *d_x_*) of this mechanical motion amplifier. The amplification factor α can be calculated as in Equations (3) and (4).

(3) α=1/tan⁡θ

(4) $$d_{x}=\alpha d_{y}$$

To change the angle inside the V-shape springs, this amplifier could achieve different amplification factors. And the fold beam inside this V-shape springs is also carefully designed. When calculated, this quad mass gyroscope characteristic, the V-shape springs cannot provide enough stiffness to make the natural frequency of the sense mode match the drive mode which is firstly confirmed about 13 kHz.

According to Hooke’s Law, the spring coefficient could be obtained by Equation (5). In this V-shaped spring, the spring coefficient in the X direction can be transferred into the Y direction as shown in Equation (6). Finally, considering the *Y*-axis as the sense direction, the equivalent stiffness of the sense direction can be calculated as Equation (7). Therefore, the fold beam can provide additional stiffness in the sense direction and match the sense mode and drive mode.

(5) $$K_{spring}=F/X\;$$

(6) $$K_{X}=\frac{F_{x}}{d_{x}}=\frac{{\frac{1}{\alpha }F}_{y}}{\alpha d_{y}}$$

(7) $$K_{s}=\alpha^{2}K_{x}+K_{y}$$

In Equation (7), *K_x_* and *K_y_* represent the stiffness coefficient of the beam connected to the V-shape springs. The angle θ should not be too big or too small. If *θ* was designed bigger than 25°, the amplification factor α would be smaller than 2, which cannot provide a satisfied effect on amplification. On the other hand, if *θ* was designed smaller than 10°, the stiffness of the X direction would be too small that the structure would be too fragile.

**Figure 4:**
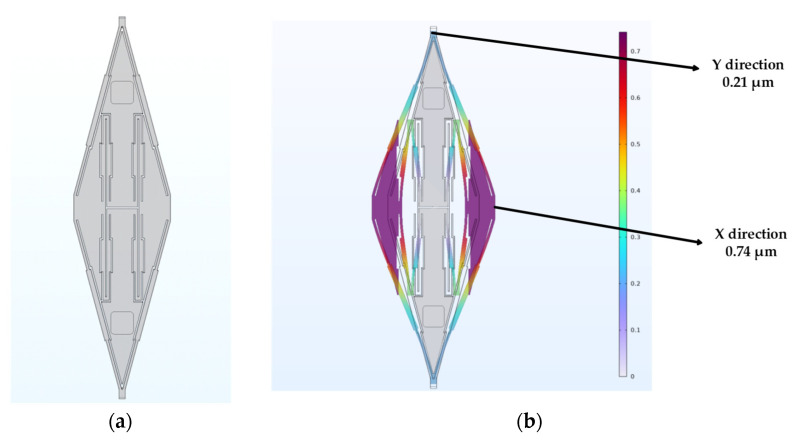
The results of steady simulation of mechanical motion amplifier. (**a**) The model of mechanical motion amplifier. (**b**) Displacement distribution of the Steady simulation.

After optimization, the angle in this work was confirmed to be *θ* = 15°. The amplification factor of the mechanical motion amplifier was also simulated under the condition of steady analysis. As shown in Figure 4, in the simulation, a force which pretended to be the Coriolis force was given to the upper and lower end of this V-shaped spring. The result showed that in this amplifier, the displacement can be changed from that induced by Coriolis to the movement of sensing capacitors and be magnified 3.65 times, which is quite the same with calculation.

### 2.4. Other Parameter Designs

The beams and the proof masses have the same thickness of 60 μm, the device size is 4 mm × 5 mm. The drive beams are four-fold beams with a width of 12 μm and length of 400 μm for each beam. The mechanical motion amplifier parameters are a width of 8 μm, length of 540 μm and angle of 15°, as discussed in Section 2.3. In the middle of the amplifier, the fold beam consists of a thinner and a thicker rectangle (Figure 4a). The thicker one’s width is 15 μm, and the length is 230 μm; this kind of composite rectangle design can precisely adjust the stiffness of the beams. This kind of design is used on almost all beams inside this QMG. More parameters about this gyroscope are as follows in Table 1.

## 3. Finite Element Simulations

We used COMSOL, a commercial finite element program, to model this quad mass gyroscope. We considered a thickness of 60 μm for the whole structure which is the same as fabrication. The material properties of the silicon were chosen to be single-crystal (isotropic). We used free tetrahedral mesh to divide the structure with the maximum units of 50 μm and minimum units of 5 μm. Using the setting above, different kinds of Finite Element Analysis were carried out to observe the characteristics of this gyroscope.

**Figure 5:**
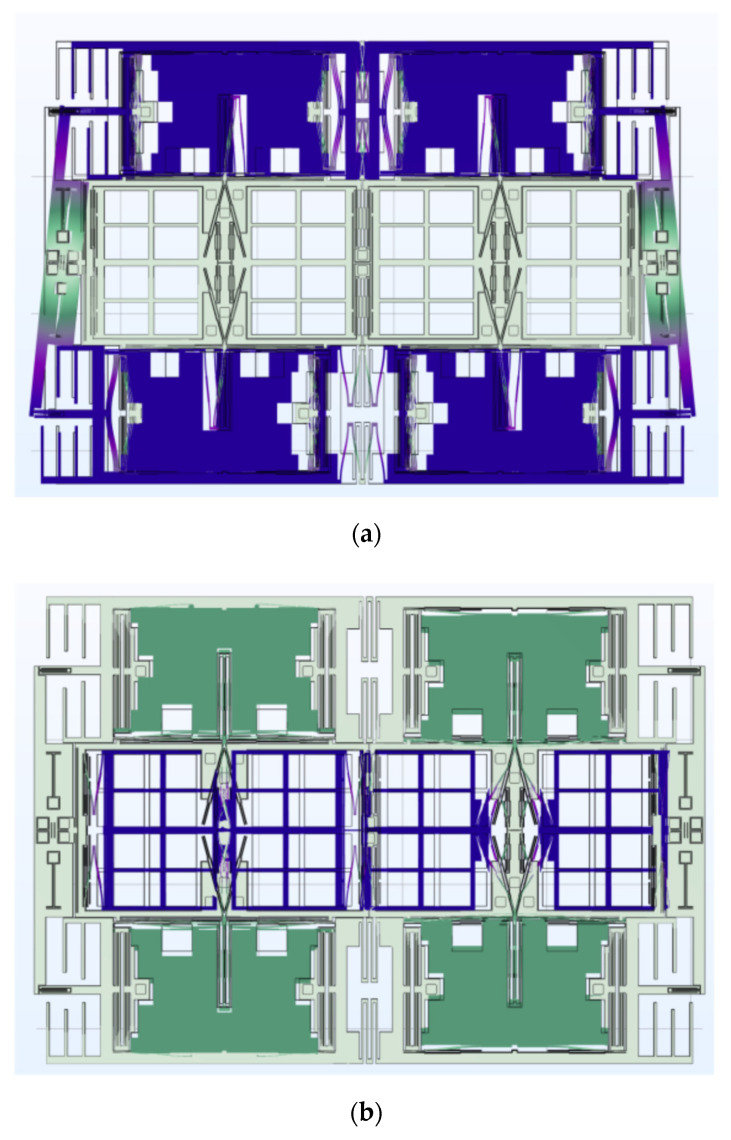
Modal analysis of gyroscope structure. (**a**) Drive mode. (**b**) Sense mode.

**Figure 6:**
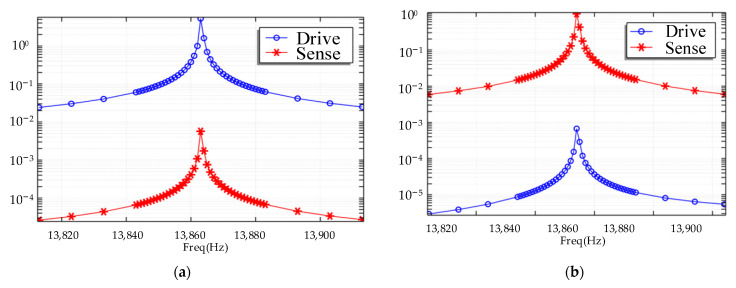
The results of harmonic response analysis. (**a**) Mechanical coupling from driving mode to sensing mode. (**b**) Mechanical coupling from sensing mode to driving mode.

First of all, the modal analysis was used to observe that the frequency of the QMG. The working modes of the gyroscope are simulated, and the results are shown in Figure 5. The modal frequency of the drive mode was 13.8 kHz and 13.9 kHz for the sense mode, which perfectly agreed with the design. Moreover, a harmonic response simulation was also carried out to check the decoupling design of the structure. In the simulation, a harmonic signal at the drive-mode natural frequency is given to the structure on the drive electrode, as shown in Figure 6a. The amplitude of the drive mode is 7.43 μm. Meanwhile, the observed amplitude of the sense mode is 5.5 × 10^−3^ μm. The amplitude ratio of the sense mode to the drive mode is 0.07%. In Figure 6b, a harmonic signal at the sense mode natural frequency is given to the gyroscope. The amplitude of the sense mode is 1.65 μm, and the amplitude of the sense mode is 0.8 × 10^−3^ μm. Then, the amplitude ratio of the drive mode to the sense mode is 0.04%. The results above show that the drive and sense movement can be literally decoupled.

**Figure 7:**
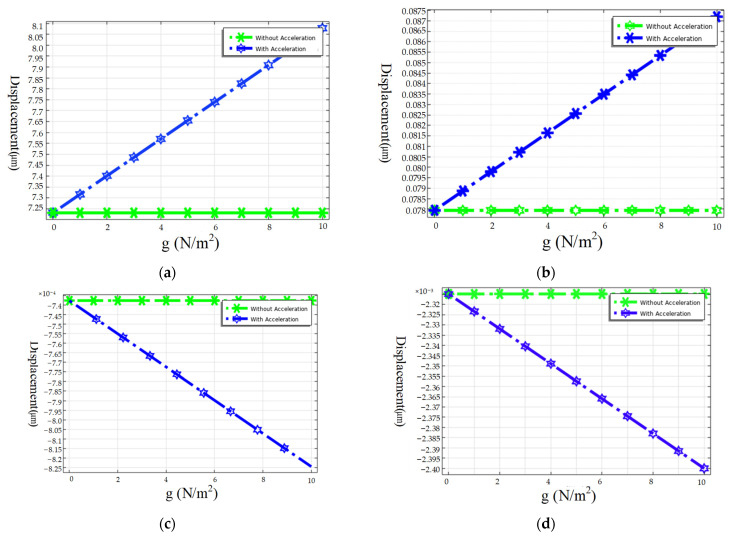
The results of linear acceleration analysis. (**a**,**b**) Drive parts’ movement under X and Y direction acceleration, respectively. (**c**,**d**) Sense parts’ movement under X and Y direction acceleration, respectively.

The influence of linear acceleration to this structure was also carried out in COMSOL as shown in Figure 7. First, the gyroscope was vibrating in the drive mode, which is its normal working state. Then, linear acceleration ranging from 1 g to 10 g was loaded onto *X*-axis and *Y*-axis, respectively. In Figure 7a,b, the relationship between *X*-axis/*Y*-axis acceleration and the drive part displacement is depicted. The rest of the pictures are similar but show the displacement of the sense part.

The simulation results showed that the acceleration had barely any effect on the change in sense capacitance. Though the drive part’s movement under *X*-axis acceleration can reach about 0.8 μm, the design of the differential sense capacitance makes sure it would not influence the output. Under other circumstances, the linear acceleration effect was calculated to be 0.03°/s/g, and this gyroscope had great resistance over linear acceleration.

## 4. Device Fabrication

The Gyroscope is divided into two parts. The device structure is fabricated with a silicon on insulator (SOI) wafer, and the CAP wafer is fabricated in a (100) orientated wafer.

**Figure 8:**
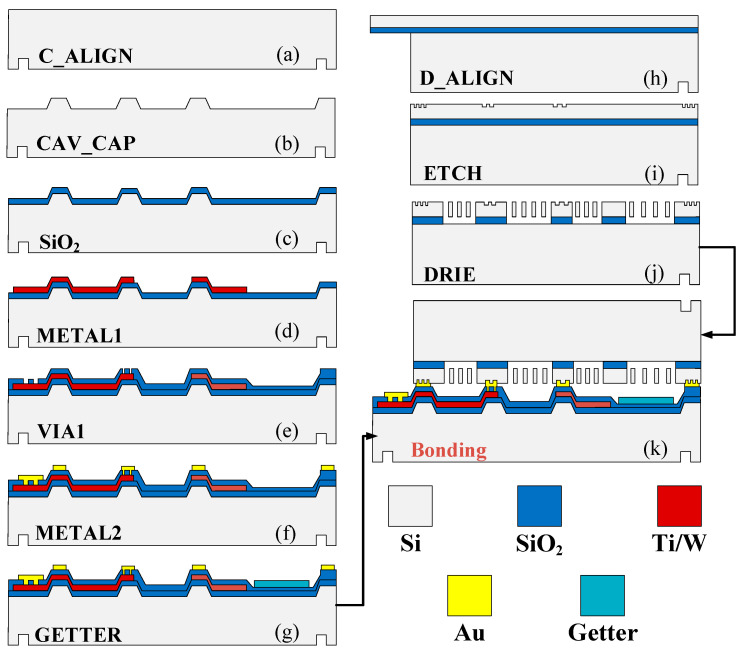
Fabrication process.

Figure 8 depicts the whole fabrication process of this gyroscope. First, the back alignment marks of the CAP wafer are defined by mask1 and the Deep RIE process (Figure 8a). Then, it used mask2 to define thermal SiO_2_ as a hard mask and etched 5 μm cavities using KOH wet etching (Figure 8b). After removing the hard mask, a 500 nm thermal oxide layer was grown as an insulator layer (Figure 8c). The first layer electrical connections are formed by metal sputtering Ti/W and wet etching of spare metal through the pattern of mask3 (Figure 8d). The insulator between the first and second metal layer is formed through PECVD and then used mask4 and SAMCO Rie to define the holes through two metal layers (Figure 8e). Finally, the bond ring and second metal layer are formed by metal sputtering and IBE with the pattern of mask5 (Figure 8f). The last step of the CAP wafer’s fabrication was depositing the getters (Figure 8g).

**Figure 9:**
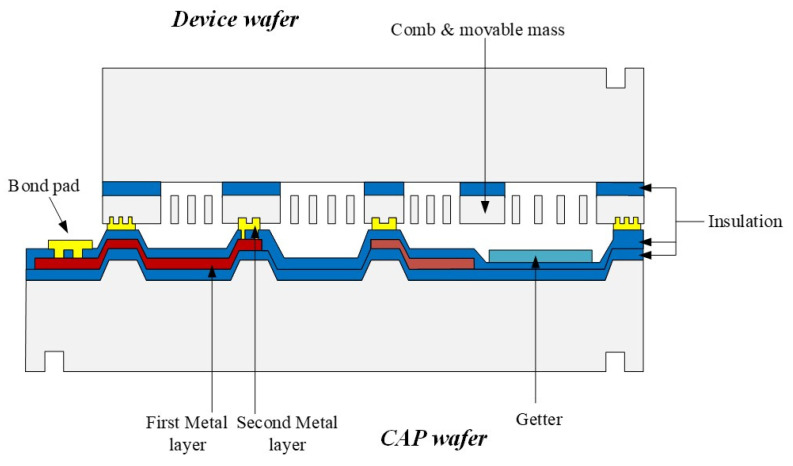
Device structure schematic.

The device wafer is fabricated using the following steps. First, the back alignment is defined with mask7. The positions of the structure which are on top of the electrode are first released from the backside of the wafer by etching the handle silicon layer through to the buried oxide layer using Deep RIE with mask8 (Figure 8h). Then, a shallow trench of about 4 μm was defined with mask9 and etched using silicon dioxide as a mask (Figure 8i). Finally, the structure is etched through to the buried oxide layer by DRIE with mask10, then released by the Vapor HF etch (Figure 8j). The device is fully fabricated after the gold silicon eutectic bonding of the CAP wafer and the device wafer. The final device is shown in Figure 9.

**Figure 10:**
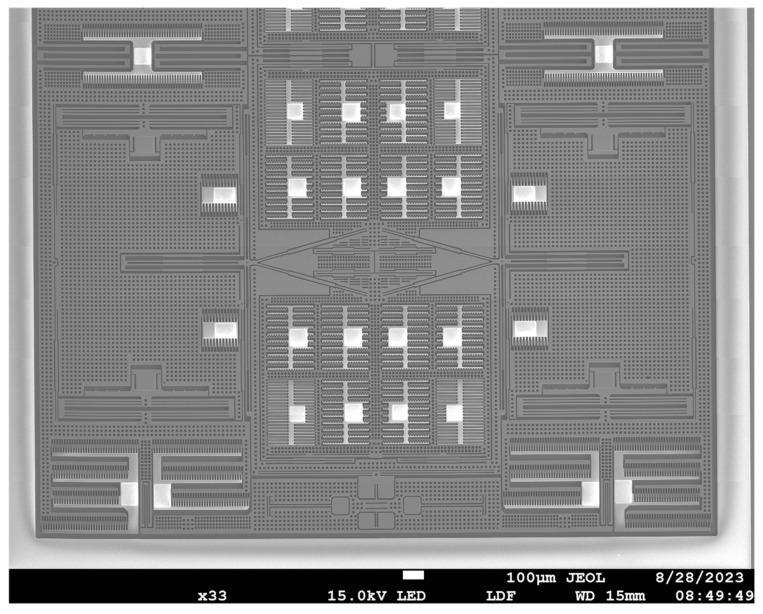
SEM image of fabricated gyroscope device wafer.

**Figure 11:**
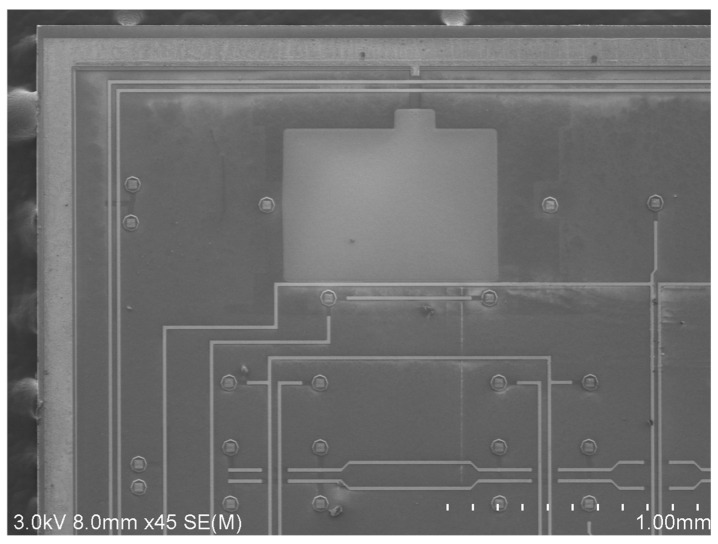
SEM image of fabricated gyroscope CAP wafer.

The SEM images of the fabricated device are shown in Figure 10 and Figure 11. Due to the device limitations, only half of the QMG can fit into an image. As Figure 10 shows, the drive capacitance finger length and overlap length were 42 μm and 4 μm, respectively. The sense capacitance finger length and overlap length were 18 μm and 2 μm, respectively. They were both fabricated as designed. However, the finger gap was 0.4 μm larger than designed because of the Deep RIE CD loss. The SEM image of the CAP wafer was shown in Figure 11. The width of the metal wire on top and the bonding ring was 20 μm and 120 μm, respectively. All of the key dimensions of the CAP wafer were closely matched with the design values.

## 5. Test Results

### 5.1. Frequency Response Test

**Figure 12:**
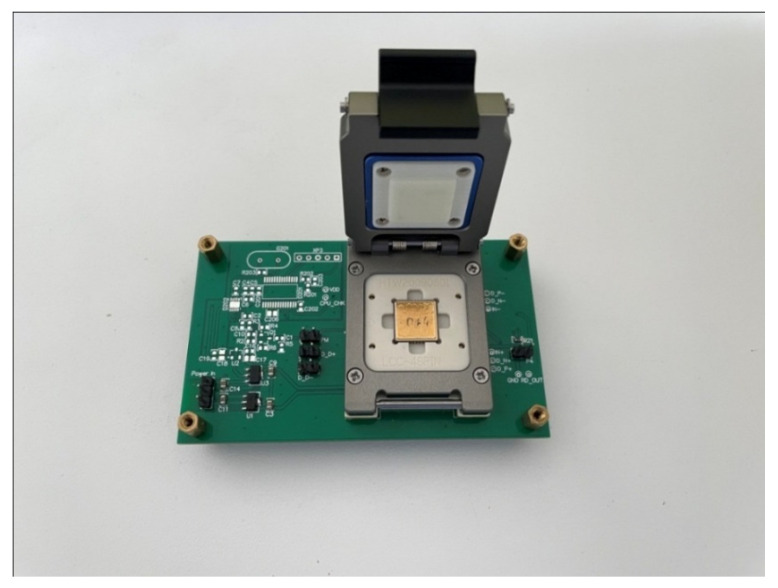
Photo of the experimental PCB.

The frequency response and ring-down tests were performed in order to characterize the basic parameters of the QMG. The experimental circuit is shown in Figure 12. In order to minimize the influence of parasitic capacitance and external environment on weak capacitance signals, the Gyroscope is encapsulated separately into the ceramic package. Therefore, the sensor is in a relatively stable environment. Then, the frequency response test was carried on.

**Figure 13:**
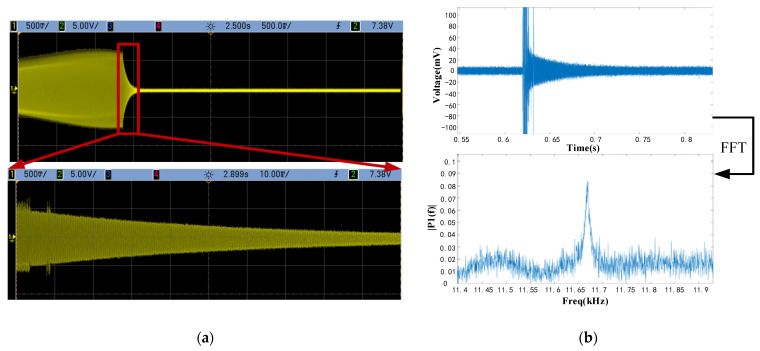
Results of ring-down test. (**a**) Ring-down test result on oscilloscopes, (**b**) ring-down data on MATLAB and FFT analysis of ring-down data.

To precisely get the ring-down signals, we use a capture board with a maximum sampling rate of 250 khz. During the test, the sample rate was set to be 120 kHz, which is about ten times over the frequency of the gyroscope. The sample time was set to be 3 s. The total data volume is 360,000. The ring-down part of data was extracted and replotted into curves using MATLAB R2021a, as shown in Figure 13b.

The frequency characteristics were obtained by calculating the results of the ring-down test in MATLAB using the FFT function. After calculation, the accurate resonant frequency f was shown in Figure 13b. The resonant frequency obtained from the analysis is 11.67 kHz, which is basically within a reasonable range for the results of the structural simulation. The error was mostly caused by fabrication. During the Deep RIE process, the drive beams were etched more than designed, which caused a lower stiffness in the drive mode. This resulted in a lower natural frequency.

### 5.2. Gyroscope Characteristic Testing

**Figure 14:**
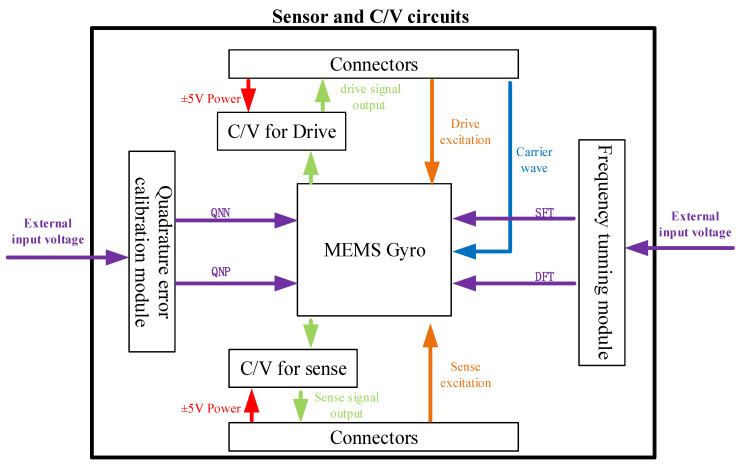
The diagram of gyroscope measurement and control circuit’s daughter board.

**Figure 15:**
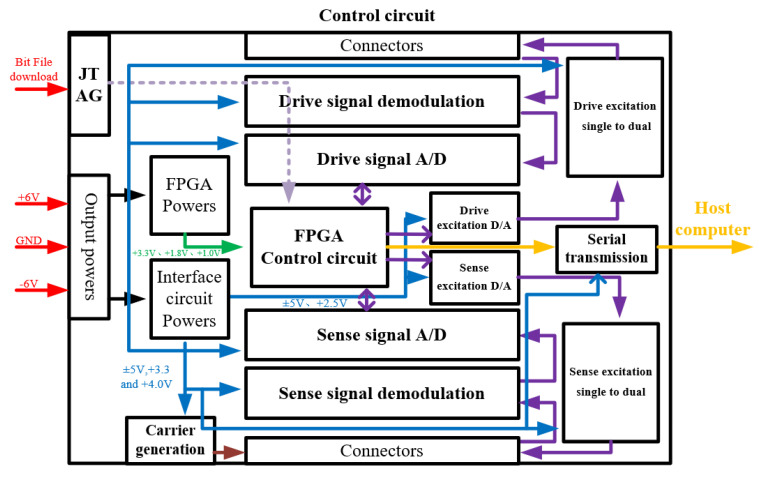
The diagram of gyroscope measurement and control circuit’s mother board.

**Figure 16:**
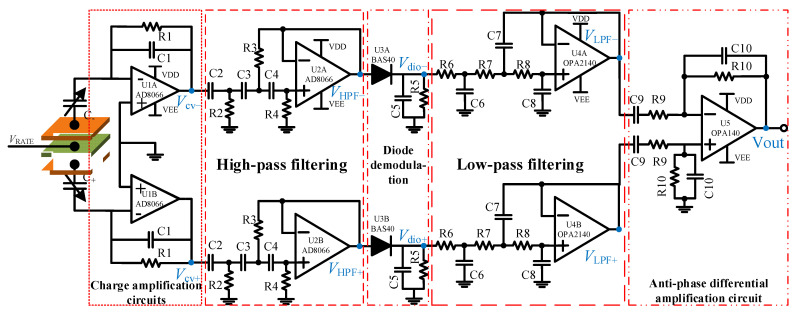
The diagram of gyroscope measurement and control circuit’s C/V analog interface circuit.

The overall gyroscope measurement and control circuits consisted of two parts. As shown in Figure 14, the first one included a MEMS gyroscope, a CV circuit, a quadrature calibration voltage module and a frequency tuning module. In Figure 15, the other one includes an FPGA control circuit, signal demodulation circuits, AD/DA circuits, excitation single-to-dual circuits, a carrier generation circuit and a voltage reference module. Among them, the design of the C/V circuit is shown in Figure 16. The C/V analog interface circuit consisted of charge amplification circuits, high-pass filter circuits, diode demodulation circuits, low-pass filter circuits and anti-phase difference amplifier circuits.

When the gyroscope was working, the drive signal was modulated in a mother board and delivered to a daughter board, then input into the device through A/D circuits. From the drive mode output signal, the vibration information of the device can be obtained and transferred from the daughter board to mother board through C/V circuits. Moreover, it was analyzed by FPGA control circuits to output a feedback drive signal. This close-loop circuit ensured that the gyroscope was vibrating under a constant amplitude, constant frequency situation.

After an angular rate input, the gyroscope sense mode was excited. The output sense signal was also transferred into the FPGA control circuit. After analyzing this signal, the FPGA will output a feedback signal to cancel the vibrations in the sense mode. In our circuits, The frequency tunning module and quadrature error calibration module were also designed to improve the performance of the gyroscope.

The C/V circuits in Figure 16 mean that the capacitance signal can change to a voltage signal. The charge amplification circuit can pick out the change in the charge change on capacitive plates and then turn it into a voltage signal. The high-pass filter circuit can filter out the carrier wave signal. Then, the diode demodulation circuit demodulates a useful signal from the carrier signal. Finally, two signals were sent to the differential amplification circuit to realize the elimination of common-mode signals and differential amplification of useful signals.

**Figure 17:**
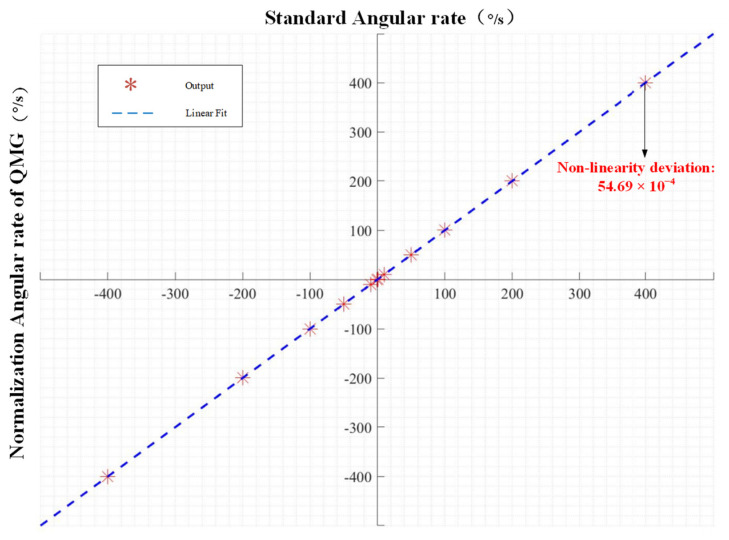
Measured angular rate output of Gyroscope. *X*-axis represents the normalized QMG response; *Y*-axis represents the angular rate of rate table. The scale factor nonlinearity is 54.69 ppm in the full range of ±400°/s.

Using the circuits mentioned above, the Gyroscope characteristic tests were carried out. The first test was about the scale factor nonlinearity. A rate table is used to apply an angular rate to the QMG so that we can get its angular rate response. Figure 17 shows the measured angular rate response of the QMG. The input angular rate is from −400°/s to 400°/s. The *X*-axis data of Figure 17 are the angular rate from the rate table, which are used as a standard angular rate. The *Y*-axis data were the angular rate output of the QMG after normalization. The scale factor nonlinearity is 54.69 ppm in the full-scale range of ±400°/s.

**Figure 18:**
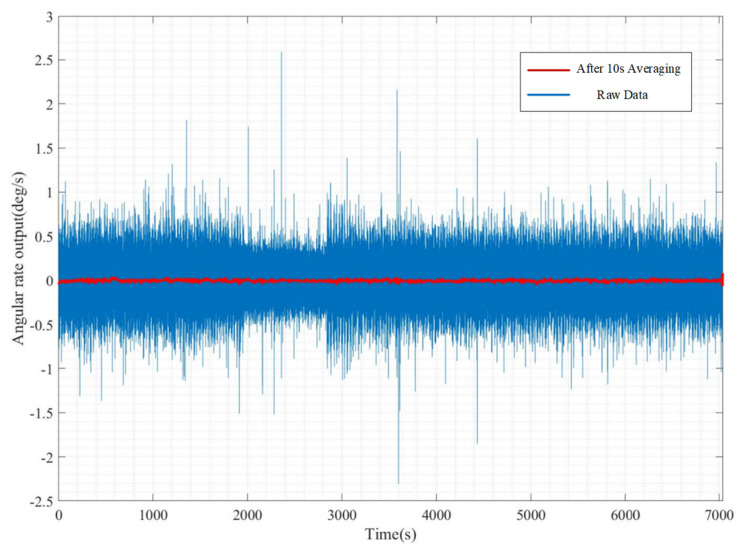
Angular rate output of the QMG system. The blue line is the raw data and the red line is the data being averaged for 10 s. After calculating, the bias stability was 44.53°/h.

The zero rate output (ZRO) data are collected for 2 h at room temperature in order to calculate the bias stability of the QMG gyroscope system. The zero rate output of the QMG under constant temperature and pressure is shown in Figure 18. In Figure 18, the blue line is the raw data of the Gyroscope output, and the red line is the data being averaged for 10 s. As Figure 18 shows, the zero rate output data had a lot of undesired signals, which may have been induced by external vibration. By calculating the standard deviation of the averaging data, the bias stability was obtained to be 44.53°/h.

## 6. Conclusions

In this paper, the design of a quadruple mass gyroscope with compliant mechanical amplification was successfully proposed, designed and fabricated. Due to the innovative mechanical amplification design, the Coriolis force signal is therefore amplified before the electric domain, which successfully enlarged the mechanical sensitivity of the QMG for 365%. The QMG can realize the decoupling of the drive mode and sense mode and is insensitive to external vibrations such as linear accelerations. Then, the gyroscope was fabricated through gold–silicon bonding of an SOI wafer and a Cap wafer. The prototype has shown a scale factor nonlinearity of 55.69 ppm. The bias stability of the QMG is calculated to be 44.53°/h. The QMG demonstrated in our work realized the combination of mechanical amplification mechanisms with the gyroscope. The mechanical amplification structure also provides a new way to design high-performance inertial sensors.

## Figures and Tables

**Table 1:** Main parameter of QMG structure.

Parameter	Drive Mode	Sense Mode
Resonant frequency	13,863 Hz	13,919 Hz
Spring constant	5590 N/m	5637 N/m
Come finger length	42 μm	7 μm
Come finger width	4 μm	4 μm
Nominal electrode overlap	18 μm	3 μm
Nominal capacitive gap	2 μm	2 μm
Number of fingers (all)	684	3812
Nominal capacitance (all)	6.54 pF	6.08 pF
Number of fingers (Drive)	492	1906
Nominal capacitance (Drive)	4.7 pF	3.04 pF
Number of fingers (Sense)	192	1906
Nominal capacitance (Sense)	1.84 pF	3.04 pF
Capacitive scale factor	0.102 pF/μm	1.01 pf/μm
Mass of proof mass	0.737 mg	0.627 mg

## Data Availability

The data presented in this study are available from the corresponding author on request. The data are not publicly available due to privacy.
